# Functionalisation of Polydimethylsiloxane (PDMS)- Microfluidic Devices coated with Rock Minerals

**DOI:** 10.1038/s41598-018-33495-8

**Published:** 2018-10-19

**Authors:** Yara A. Alzahid, Peyman Mostaghimi, Alireza Gerami, Ankita Singh, Karen Privat, Tammy Amirian, Ryan T. Armstrong

**Affiliations:** 1 School of Minerals and Energy Resources Engineering, The University of New South Wales, Sydney, NSW 2052 Australia; 2 Electron Microscope Unit, The University of New South Wales, Sydney, NSW 2052 Australia; 3 Australian School of Petroleum, Faculty of Engineering, Computer and Mathematical Sciences, The University of Adelaide, Adelaide, SA 5000 Australia

## Abstract

Fluid flow in porous rocks is commonly capillary driven and thus, dependent on the surface characteristics of rock grains and in particular the connectivity of corners and crevices in which fluids reside. Traditional microfluidic fabrication techniques do not provide a connected pathway of crevices that are essential to mimic multiphase flow in rocks. Here, geo-material microfluidic devices with connected pathways of corners and crevices were created by functionalising Polydimethylsiloxane (PDMS) with rock minerals. A novel fabrication process that provides attachment of rock minerals onto PDMS was demonstrated. The geo-material microfluidic devices were compared to carbonate and sandstone rocks by using energy dispersive X-ray spectroscopy, scanning electron microscopy (SEM), contact angle measurements, and a surface profilometer. Based on SEM coupled with energy-dispersive X-ray spectrometry (SEM-EDS) analyses, roughness measurements, contact angle, wettability, and roughness were comparable to real rocks. In addition, semivariograms showed that mineral deposition across the different geo-material devices was nearly isotropic. Lastly, important multiphase flow phenomena, such as snap-off and corner flow mechanisms, equivalent to those occurring in reservoir rocks have been visualised. The presented approach can be used to visualise rock-fluid interactions that are relevant to subsurface engineering applications, such as hydrocarbon recovery and CO_2_ sequestration.

## Introduction

Multiphase flow is relevant to several industrial fields, such as geologic CO_2_ sequestration^1–4^, enhanced oil recovery^5–7^, hydrology^8,9^ and fuel cells^10^. Wettability, which is the preference of a fluid to a solid, is a key parameter that influences multiphase flow in porous media^11–15^. Wettability of surfaces is commonly quantified through contact angle (θ) measurements where θ < 90° corresponds to water-wet surfaces and θ > 90° corresponds to oil-wet surfaces^11,16^. Flow mechanisms that occur between rock grains are highly dependent on wettability, such as snap-off^17^ and corner flow^18^, and are of particular interest for carbon capture and storage^19^ and enhanced oil recovery technologies^5–7^. Hence, visualising these processes in an environment similar to that of rock grains that provides contact angles and flow mechanisms equivalent to those found in reservoir rocks is essential to the design of any microfluidic device and the subsequent study of the fundamental physics that occur within the space between rock grains.

**Figure 1:**
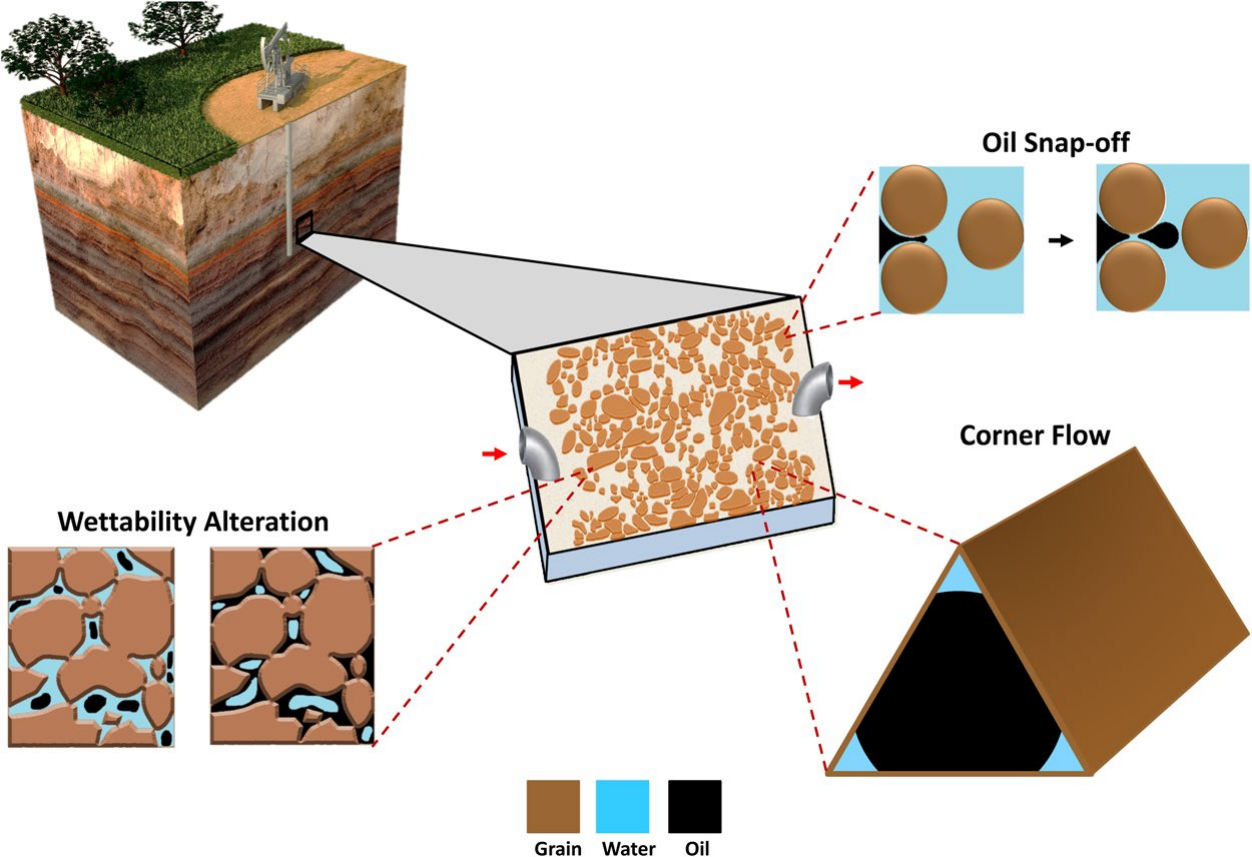
Geo-material microfluidic chip (centred) and its applications/features. This figure highlights the ability to re-create the reservoir rock environment, due to the added minerals on the grains and pore space of the microfluidic platform. This type of microfluidics devices can capture several important rock/fluid features, including: corner flow, wetting conditions (oil or water-wet), and snap-off.

Snap-off and corner flow are responsible for disconnection and trapping of hydrocarbons and/or CO_2_. Snap-off occurs when a droplet of non-wetting phase (typically oil or gas) disconnects, leading to the trapping of that phase (Fig. 1). The basic physics of snap-off was first described by Rayleigh^20^. Flow instabilities arise due to geometrical changes, resulting in differences of capillary pressure. For a jet, as described by Rayleigh^20^, with a given wavelength, regions of constriction develop, leading to higher capillary pressures than adjacent regions. This high capillary pressure causes fluids to flow away from the constriction eventually resulting in the collapse of an interface. For a porous media, a similar situation occurs when an oil droplet spans many pores. The leading and trailing ends of the oil droplet have a given capillary pressure, while the pore throat constriction between pores can result in a larger capillary pressure due to the swelling of a wetting film in the neck region. Hence, the formation of snap-off is also largely dependent on the specific pore geometry, i.e. pore-wall curvature, and pressures in the wetting and nonwetting phases^21,22^. For a complete water-wet environment (θ = 0), and at capillary-dominated flow regime, snap-off favours a large aspect ratio (pore radius/ throat radius) during an imbibition process, such as water flooding^5,23–25^. In pores of rocks with complex pore geometry, snap-off is controlled by corner flow (Fig. 1), which is the advancement of the wetting phase through connected water films that cover the water-wet grains. This mechanism was proposed by Ransohoff, *et al*.^26^ as the quasi-static snap-off criterion, which predicts non-wetting phase snap-off within a smoothly constricted noncircular capillary.

In water-wet rocks, thin films of water cover the surfaces of grains and extend across adjacent grains. These films influence immiscible displacement. For example, in enhanced oil recovery processes and during imbibition, water is known to flow in the pore space via the wetting films ahead of the advancing meniscus, bridging across the pore space containing oil, which creates the possibility for snap-off events and influences the order in which pores are saturated. Pore network models are often used to demonstrate the importance of corner flow during immiscible displacement^27^. Other parameters such as aspect ratio^28^, flow rate^29^, contact angles^7,30^ and dimensionless parameters (such as capillary number and viscosity ratio)^28,31–35^ have also been investigated using pore-network models. Pore network models that do not account for corner flow provide significantly different results than models that allow for the wetting phase to reside in the corners of the network^31^. Therefore, when considering idealised geometries for experiments, it is essential to consider systems that support the natural mechanisms of porous media flow.

Throughout the past few decades, microfluidic devices with porous media patterns representing porous rock have been used to study fluid flow for subsurface engineering^6,36–39^. Images or 3D models of actual porous rock can be generated using micro-computed tomography (micro-CT), focused ion beam-scanning electron microscope (FIB-SEM) or nuclear magnetic resonance (NMR)^36,40–42^ methods. These images can then be transformed to a microfluidic chip using standard photolithography or soft photolithography^42^. Karadimitriou and Hassanizadeh^43^ summarise microfabrication methods for micromodels and the history of their usage in soil science and petroleum research, particularly enhanced oil recovery (EOR)^6,36,38,44–46^. However, microfluidic models generally do not allow for the connectivity of corners and crevices due to their 2D nature, in addition to their lack of roughness, which can limit the type of flow processes that occur during immiscible displacement experiments.

Several fabrication methods for geo-material chips have been proposed to produce microfluidics devices that mimic real rock. Song, *et al*.^37^ developed an approach to study acid injection in carbonate formation by etching microfluidics channels into a natural calcite rock, which required multiple pre-processing steps. Song and Kovscek^47^ developed a method to create a functionalised 2D silicon micromodel with pore surfaces coated mainly with kaolinite clay, which allows for the study of fluid–solid interactions. In addition, these authors published another paper describing the use of their functionalised microfluidic platform to study the response of clay to low salinity brine injection^48^. Other microfluidic device modifications that aid in representing real rock include altering the wettability of the porous network. Lee, *et al*.^49^ developed a novel method termed “Stop-Flow-Lithography (SFL)” to fabricate microfluidics devices with controlled wetting properties in a single lithographic step. They showed immiscible fluid displacement in single pore bodies with varying wetting properties. Porter, *et al*.^50^ developed a novel fabrication method whereby fractures are etched into thin sections of different rock types (shale, sandstone, and siltstone) by a custom-built femtosecond laser direct-write (LDW) system. Similarly, Gerami, *et al*.^51^ presented a coal geo-material microfluidic chip that was fabricated by etching a fracture pattern into a coal surface using three-dimensional laser micromachining. Wang, *et al*.^52^ demonstrated a novel way to coat a controlled thickness of calcite (CaCO_3_) nanocrystal layer onto a simple glass microfluidic channel. This process was achieved by converting the inner surface of the microfluidic silica channels to CaCO_3_ through numerous chemical surface modifications to functionalise the glass surface to grow a CaCO_3_ nanocrystal layer. Singh, *et al*.^53^ used another approach to design a microfluidic device of a sandstone rock called “Real Rock Microfluidic Flow Cell” (RRMFC). They mounted a thin section (500 μm) of a sandstone rock between two covers that were bonded via a plasma generator. Lastly, another approach taken by Zhu and Papadopoulos^54^ demonstrated two-phase flow in transparent miniature packed beds with rough grains and analysed the effect of roughness on flow.

Traditionally, core-flooding experiments are conducted to establish the wettability, capillary pressure data, relative permeability curves, and oil recovery for a given reservoir rock sample^52^. With different geo-material microfluidics, or rock-on-a-chip methods, oil recovery can be measured and pore-scale mechanisms (on the order of micrometres) can be captured. EOR mechanisms such as chemical or low salinity can be tested prior to core scale experiments to study a wide range of the parameter space necessary to understand pore-scale oil recovery mechanisms and/or evaluate screening criteria for particular EOR applications. This can guide the planning and assessment of core floods and/or illustrate if a given EOR mechanism could suppress oil snap-off, enhance dissolution and/or cause fines migration for a given set of flooding conditions.

The aforementioned fabrication methods of geo-material microfluidics devices involve several pre-processing and complex fabrication steps, in addition to the use of expensive machines and/or packed grains that obstruct visualisation. In addition, the previous works have not provided evidence that the essential pore-scale flow mechanism of corner flow leading to snap-off actually occur in the fabricated geo-material chips. We propose a simple process for functionalising the pore space of PDMS microfluidic chips with rock minerals and controlled wetting conditions, which allows for snap-off and corner flow mechanisms to occur. The resulting geo-material microfluidic device allows for direct visualisation and understanding of rock-fluid interactions in oil reservoirs during any type of flooding. We describe the coating of PDMS microfluidic chips with the following materials: (1) quartz and kaolinite to represent sandstone^55^ formations, and (2) calcite to represent carbonate formations^56^. We characterise the surface composition, roughness, wettability, and uniformity of the functionalised PDMS models and compare them with reservoir rock samples. Lastly, we conduct water flooding experiments to study the resulting pore-scale flow mechanisms and conclude that many of the well-established mechanisms important for porous media flow are captured by the microfluidic device due to the wettability and connectivity of corners and crevices added by the geo-material coating process.

## Results and Discussion

### PDMS Surface modifications for generating geo-material microfluidic chips

**Figure 2:**
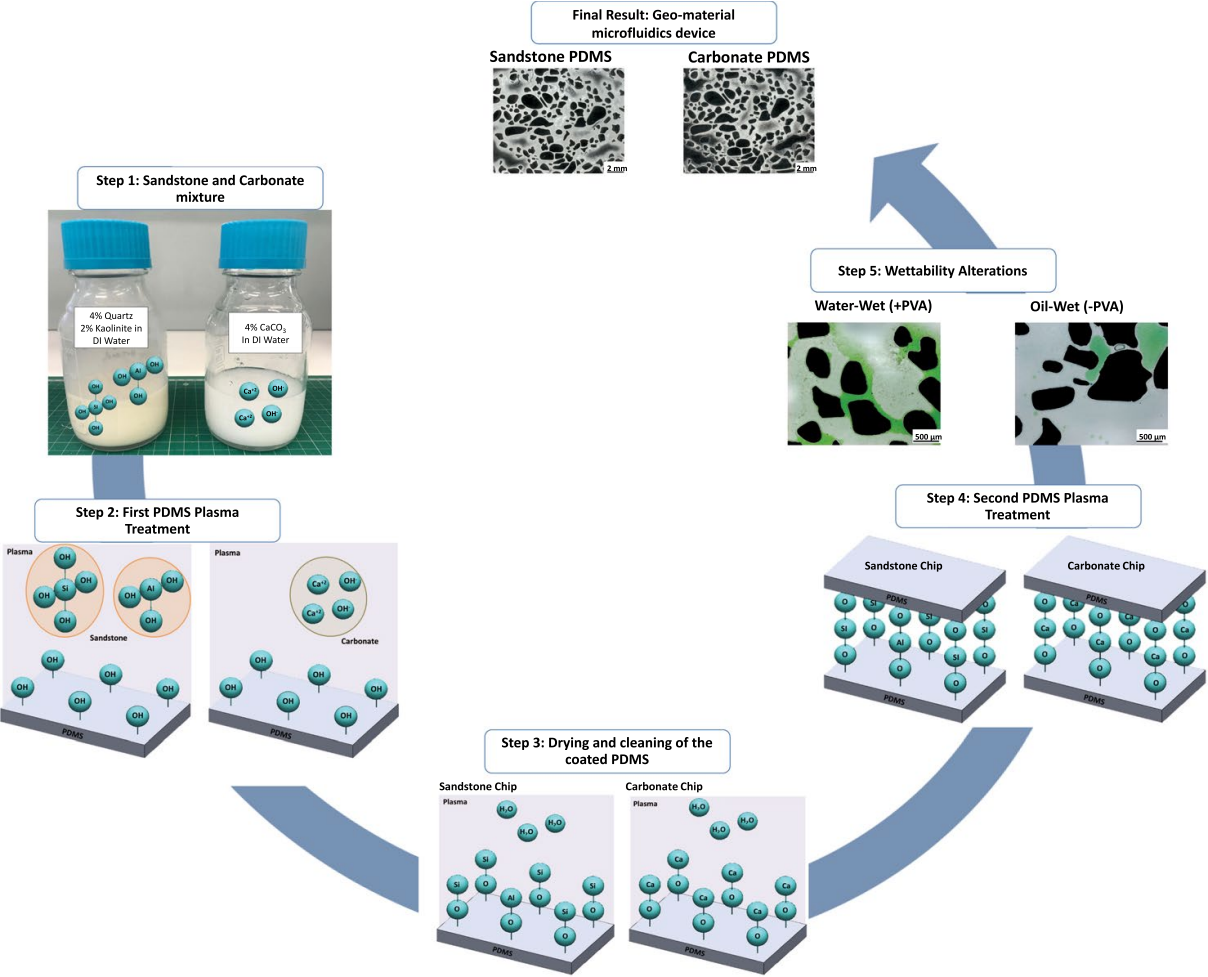
The workflow for fabrication of the geo-material PDMS microfluidics device. The sandstone and carbonate mixtures are first prepared by mixing quartz and kaolinite in DI water, and calcite in DI water, respectively. When mixing quartz and kaolinite with DI water, it mainly results in soluble silica (SiOH_4_), also referred to as silicic acid, and kaolinite gibbsite (AlOH_3_). As for the calcite mixture, the calcium carbonate does not mix well with water, as it rapidly separates when left stagnant, however, some calcium ions separate and can attach with silanol group in subsequent steps. When the two PDMS slides with the porous media are exposed to oxygen plasma, it attaches silanol groups on the PDMS surface, rendering it water-wet temporarily, these silanol groups then bond with the silicic acid and kaolinite gibbsite in the sandstone mixture, creating very strong siloxane bond and Al-O-Si bonds. As for the calcium hydroxide, it also attaches to the silanol groups. Then, both chips are air-dried and cleaned with Ethanol. After ensuring the pillars in the PDMS chips are clean, the PDMS with the deposited materials and PDMS slabs (covers) undergo a second plasma treatment, creating additional SiOH groups on both PDMS slides, and immediately put in conformal contact, which results in a sealed geo-material PDMS chip. Wettability alterations can then be made by adding 1% PVA solution immediately after the second plasma treatment, which makes the geo-material water-wet. Without PVA treatment the geo-material PDMS device is oil-wet.

The process of treating PDMS with oxygen plasma is well-understood^57–59^. On exposure to oxygen plasma, silanol groups (Si–OH) are added to replace –CH_3_ on the PDMS structure, which comprises of –O–Si(CH_3_)_2_− repetition units prior to plasma treatment. The Si–OH groups are then able to bond with other functional groups, such as –OH, –COOH, and ketone, when the two surfaces are brought into contact^57–61^ as depicted in Fig. 2. Plasma treatment of PDMS is crucial to functionalise the surface for depositing sandstone and carbonate mineral mixtures. In the quartz and kaolinite suspension, the presence of silicate oxygen groups in the quartz and kaolinite and hydrogen groups along the edge of the kaolinite particles are the main active groups that bond with silanol groups on the PDMS surface. Both oxygen atoms and hydroxyl groups in the quartz and kaolinite suspension can hydrogen bond with the hydroxyl groups at the ends of the PDMS chain. As for the calcite mixture, due to its suspension in water, the hydroxyl groups and calcium ions can also bond with silanol groups on the PDMS. Figure 2 highlights the bonding of chemical structures for both the sandstone and calcite suspensions with silanol groups present on the PDMS surface. Further details on fabrication are discussed in the Methods section.

### Minerals Coating Characterisation

**Figure 3:**
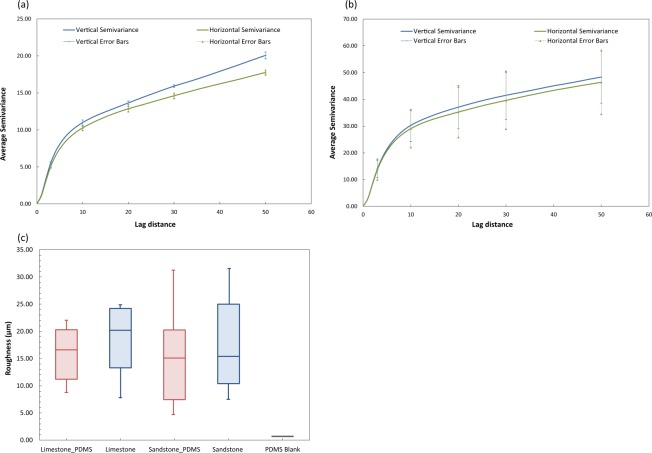
(**a**) Average semivariance of the sandstone PDMS chip. (**b**) Average semivariance of the carbonate PDMS chip. The error bars in a and b indicate the variation in average semivariances for a lag distance of 3, 10, 20, 30 and 50 pixel distance for the mineral deposited on three sandstone images and three carbonate images, respectively. (**c**) Box plot with surface height profile measurements for target areas of x = 1418 µm, y = 1000 µm for PDMS coated with carbonate (or limestone) mixture, limestone rock, PDMS coated with the sandstone mixture, sandstone rock and blank PDMS.

To characterise the functionalised PDMS surface, we used semivariograms to study the spatial arrangements of the deposited minerals and compared SEM-EDS measurements to equivalent measurements taken from reservoir rock samples. The semivariograms in Fig. 3a and b illustrate the average semivariance of the mineral deposition for both sandstone and carbonate PDMS chips, respectively for a lag distance of up to 50-pixel values (333.34 µm, scale of 0.15 pixels/µm) in both the horizontal (0°) and vertical (90°) directions. The semivariances in both samples show the expected trend of increasing semivariance with increased lag distance. The semi-variances increase with pixel distance (or lag distance) because pixels adjacent to each other are more correlated to each other than pixels far apart. The error bars in Fig. 3a and b indicate the variation in average semivariances for a lag distance of 3, 10, 20, 30 and 50 pixels for the minerals deposited on three independent sandstone and carbonate chips. For sandstone (Fig. 3a), the error bars demonstrate that there were measurable differences in the horizontal and vertical directions. This occurred because the measured variance between tested locations on the PDMS sandstone chip are relatively small resulting in narrow error bars. Conversely, the error bars for mineral deposition in the case of carbonate (Fig. 3b) are wider, which demonstrates that the variation between independent samples was greater than that for the sandstone PDMS chip. Therefore, for the carbonate PDMS chip, there were no measurable differences in the semivariances in the horizontal (0°) and vertical (90°) directions. Overall, for both sandstone and carbonate depositions, the semivariances in the horizontal (0°), and vertical (90°) directions are similar suggesting that the deposition was nearly isotropic. Isotropic deposition is an important factor for mineral deposition since wetting films are likely to be established in the deposited minerals and a directional bias of wetting films could influence experimental results.

The surface roughness data of the geo-material chips are calculated and compared to roughness values measured for carbonate and sandstone rocks. The data are obtained from the surface height profiles of multiple locations on each sample. In total, 15 locations of roughness measurements for carbonate PDMS and rock, 21 locations for sandstone PDMS and 19 locations for sandstone rock are collected. The results indicate similar roughness data for the pore surfaces of the fabricated chips and the real rock surfaces. For instance, only 9.9% variation exists between the roughness of the sandstone PDMS chip and sandstone rock. For the carbonate coated chip the variation of roughness from the real rock is 14.7%. The box plot in Fig. 3c confirms similar roughness data for the pore surfaces of the fabricated chips and the rock surfaces. For both sandstone and carbonate rocks and functionalised PDMS models, the median values (middle lines in the box plots) of roughness are similar. In addition, the roughness of a blank PDMS chip was also evaluated and the roughness data are staunchly different than functionalised PDMS (Fig. 3c). One limitation from this analysis is that these results are only valid for the rocks examined. Different roughness profiles could occur when comparing the functionalised PDMS surface to other sandstone and carbonate rocks.

**Figure 4:**
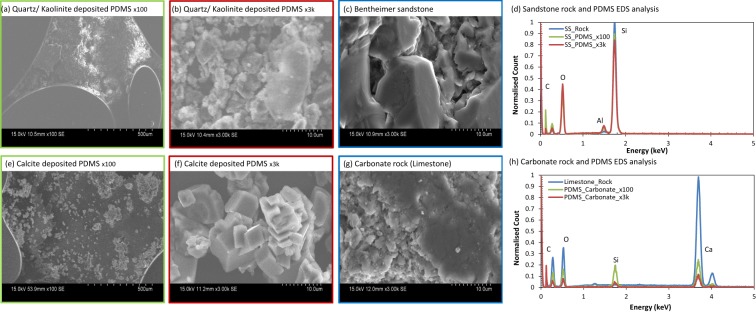
SEM images and EDS analysis of sandstone rock and PDMS (**a**–**c**), and carbonate rock and PDMS (**e**–**h**). The sandstone and calcite deposited PDMS show similar morphology and compositional analysis compared to their corresponding rocks. The image border colours in (**a**–**c**) and (**e**–**g**) correspond to the lines present in **d** and **h**.

SEM images of PDMS chips containing mineral depositions (x100) and a higher magnification (x3k) image of the relevant rock slice are present in Fig. 4. Images of the PDMS containing the minerals of interest (Fig. 4a,b,e and f) can be comparable with the rock image (Fig. 4c and g), as the particle morphologies are similar. Furthermore, EDS elemental analysis was performed on the rock slices and PDMS microfluidics chips. Normalised EDS results for the two samples provide comparable results with their respective rock types (Fig. 4d and h). However, the carbonate rock and calcite-PDMS do not exhibit exact elemental spectra in Fig. 4h, due to the presence of silicon in the PDMS backbone. In the case of the calcite-PDMS and limestone rock, the calcium peaks are similar (Fig. 4h). Carbon is present in both rock and geo-material PDMS chips due to the required carbon coating for SEM imaging. Overall, the surface chemistry appears to be similar for the functionalised chips and their reservoir rock equivalents.

### Establishment of Surface Wettability

**Figure 5:**
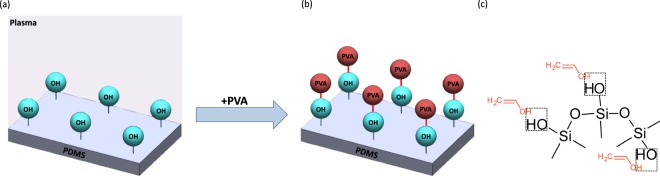
Mechanism of PDMS wettability alteration by adding PVA. (**a**) shows the PDMS surface following plasma treatment with silanol groups on the surface (Si-OH). (**b**) shows PVA bonding to the OH groups, and (**c**) shows the detailed molecular hydrogen bonding between PVA (red) and the PDMS backbone (black). The dashed lines in (**c**) show the locations of these hydrogen bonds.

**Figure 6:**
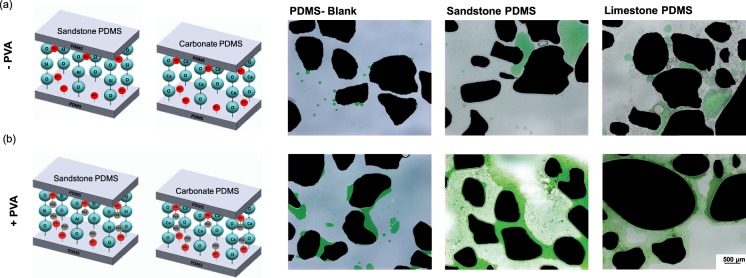
Wetting films during the two cases: (**a**) without PVA (−PVA), and (**b**) with PVA (+PVA) treatment. The cartoons on the left illustrate the molecular interactions occurring in each scenario, and the microscope images show the wetting films in each of the three PDMS chips (blank, sandstone and limestone) with and without PVA. Oil is transparent and connate water is green.

PDMS is strongly oil-wet with contact angles >100°^57–59,61,62^. A plasma treated PDMS surface temporarily creates alcoholic hydroxyls (C–OH), silanols (Si–OH), and carboxylic acids (COOH)^57,59^ that render the surface water wet for a limited period of time. As reported in Trantidou, *et al*.^61^, applying PVA to a plasma treated PDMS surface creates hydrogen bonds between the PVA molecules and the plasma treated PDMS surfaces. These hydrogen bonds on the PDMS form permanent water-wet surfaces rather than the temporary treatment created by only plasma treatment (Fig. 5). The PDMS surface treatment method developed by Trantidou, *et al*.^61^ was applied to our fabricated geo-material chips. Several tests were conducted to understand how the method influences the wettability of PDMS and the deposited minerals. Sandstone, carbonate and un-deposited PDMS chips were treated with PVA immediately after plasma oxidation, as described in Trantidou, *et al*.^61^ and imaged after 3 days to eliminate the effect of plasma oxidation. PDMS with and without PVA treatment were first subjected to connate water flooding (green), then decane oil (transparent) and left to equilibrate for 10 minutes. The three chips are shown in Fig. 6. We observe connected thin films of connate water (green) in the geo-material PDMS chips of sandstone and carbonate with PVA treatment (Fig. 6b), which demonstrates that these chips are water wet. Alternatively, without PVA treatment (Fig. 6a), PDMS is highly oil wet in all three conditions. As explained previously, the reason that PVA can alter the wettability of PDMS is that it irreversibly adsorbs onto the hydrophobic polymer films of PDMS, which alters its chemistry by the formation of hydrogen bonds between PVA and the activated PDMS surface^61^. As observed in the EDS and SEM results, regions of the functionalised PDMS chips are not completely covered with rock minerals (Fig. 4) and thus, the exposed PDMS still influences the surface wettability.

Trantidou, *et al*.^61^ performed a longevity study on their PVA treatment, and the contact angle was stable at 30–50° for 9 days, which confirms the validity of this method and long-term effect of PVA with PDMS. It is also important to note that PVA treatment on the un-deposited PDMS chip showed less connected connate water as compared to the carbonate and sandstone models. This can be attributed to the surface roughness of the geo-material chips, which allows for connate water to exist in the corners and crevices of the chip. The connate water appears to be connected since wetting films in Fig. 6 are seen to extend across grains. This allows for the establishment of connate water films throughout the microfluidic chip. The thin connate water films present for water-wet conditions of the sandstone and carbonate conditions with PVA treatment are essential for visualising pore-scale phenomena in subsurface fluid flow, as demonstrated in the next subsection.

**Figure 7:**
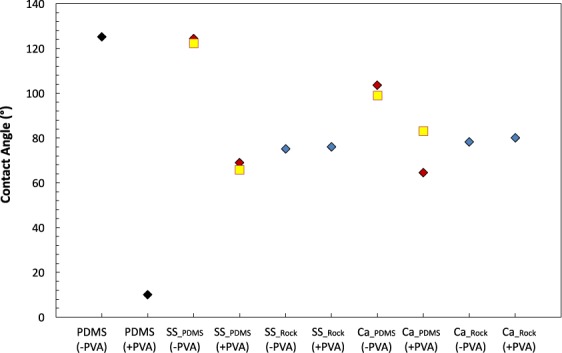
Contact angle measurements of (1) PDMS, (2) the two different geo-material PDMS chips, and (3) their respective rocks without PVA (−PVA) and with PVA (+PVA). The colours of the points correspond to the different materials present. PDMS is shown as black, geo-material PDMS is red, rock slices are blue and Cassie’s equation estimates for the geo-material PDMS is yellow.

To confirm that PVA treatment of the geo-material PDMS chips does not alter the surface wettability of the deposited grains, we compare contact angles of the rock samples to geo-material PDMS chips with and without PVA. The steps for measuring contact angle are provided in the Methods section. Contact angle measurements in Fig. 7show that PVA has no effect on the reservoir rock, as the contact angles remain in the same range (75°–80°) with and without PVA treatment. However, the geo-material PDMS chips without PVA show a highly hydrophobic surface (θ > 100°), which demonstrates that the PDMS backbone is influenced by PVA treatment, thereby controlling the contact angle, hence the oil-wet nature of the chips. Conversely, geo-material PDMS chips with PVA treatment show similar contact angle measurements to those present for the reservoir rock samples.

Cassie’s equation (Eq. 7) was used to explain the shift in the contact angle in the geo-material PDMS microfluidics devices with and without PVA treatment. Cassie’s equation provides an apparent contact angle by considering chemically heterogeneous surfaces with regions that have distinctly different contact angles^63–65^. For the functionalised PDMS surface, there are regions of exposed PDMS (−PVA or +PVA) and regions of rock minerals. The surface fractions of mineral and exposed PDMS regions are measured from microscope images. The contact angles for these two regions are taken from Fig. 7, as PDMS (−PVA or +PVA) and Ca_rock_ (−PVA) or SS_rock_ (−PVA). From Fig. 7, the contact angle measurements determined from Cassie’s equation (yellow) are in agreement with the experimentally measured contact angles in both geo-material microfluidic chips (red). This indicates that the fraction of PDMS present on the surface influences the wettability, especially in the case of no PVA treatment (−PVA).

### Flow Visualisation using the Geo-material PDMS chips

**Figure 8:**
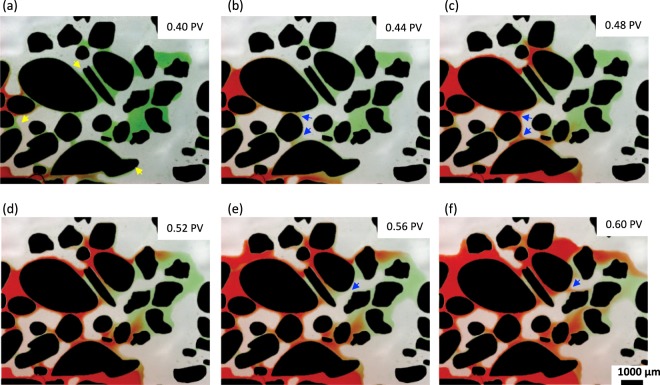
Wetting films of connate water (green) and injected DI water (red) displacing decane (transparent) indicated in yellow arrows and oil snap-off event indicated in blue arrows. From the image, swelling of connate water is observed in (**a**–**c**) and one snap-off event is depicted in the blue arrows (**e**–**f**).

With the functionalisation of PDMS with sandstone and carbonate particles, we have established: (1) a geo-chemical environment and roughness profile that is similar to a real rock, and (2) similar wetting profiles, due to the PVA treatment that alters the wettability of PDMS to water-wet but does not alter the geo-material wettability. These factors create a platform for studying the pore-scale flow. In this section, we demonstrate the swelling of connate water wetting films and snap-off in our functionalised chips. Roof^17^ describes oil snap-off as the formation of an oil droplet due to oil being pushed through a water-wet constriction into a water-filled pore. Snap-off is strongly dependent on wettability, pore scale geometry, and roughness of the pore–solid interface^25,26,66^. Figure 8 illustrates the swelling of connate water films (green) when DI water (red) is injected into a functionalised chip at connate water (green) equilibrium. We can observe that the injected water travels within the pre-existing connate water films, leaving oil behind (Fig. 8).

**Figure 9:**
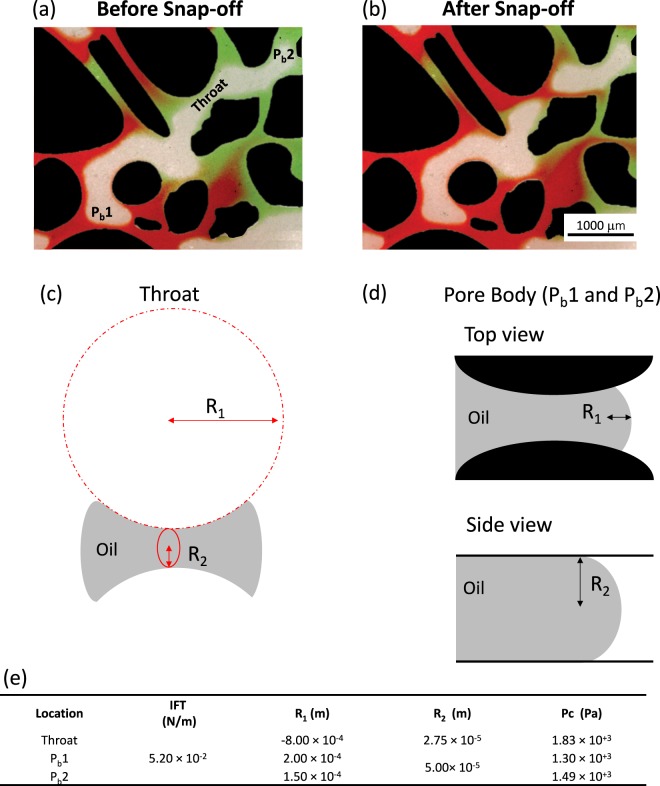
Detailed snap-off event visualisation. (**a**) Before snap-off event at 0.56 PV. (**b**) After snap-off at 0.60 PV. (**c**) R_1_ and R_2_ measurements taken for throat, and (**d**) pore bodies (P_b_ 1 and 2). (**e**) Capillary pressure measurements at throats and pore bodies (P_b_). Grains are present in black, red is DI water, green is connate water, and oil is transparent and/or grey.

As previously mentioned, snap-off is highly dependent on the specific pore geometry, i.e. pore-wall curvature, and pressures in the wetting and nonwetting phases^21,22^. Capillary pressure differences between the leading and tailing ends of an oil droplet and pore necks can be measured directly from microscopic images^67^. In our experiments, time-lapse images were taken in one location while injecting DI water (red). From the collected images, one snap-off event was observed, along with several oil clusters that were a result of previous uncaptured snap-off events, in Fig. 8(b–f). Figure 9 highlights one snap-off event at 0.56 and 0.60 PV, which correspond to before and after snap-off, respectively. Capillary pressures at different locations (throat and pore bodies) are also shown in Fig. 9. For the example snap-off event, capillary pressure in the pore throat region is greater than capillary pressures measured in the leading and tailing ends of the oil droplet. Higher capillary pressure in the pore throat region would cause the oil phase to flow away from the throat constriction and thus, leading to the collapse of an interface, i.e. a snap-off event. Capillary pressures are determined by using the Young Laplace equation, as explained in the Methods section. The two principals of curvatures are measured from the oil interface in the throat and pore bodies as visualised in Fig. 9c. The resulting capillary pressures are reported in Fig. 9d showing that pressure was larger in the throat region than the two pore bodies. This difference in pressure causes snap-off in the throat, as oil flows away from the throat region.

### Minerals Attachment Stability of the Geo-material PDMS chips

**Figure 10:**
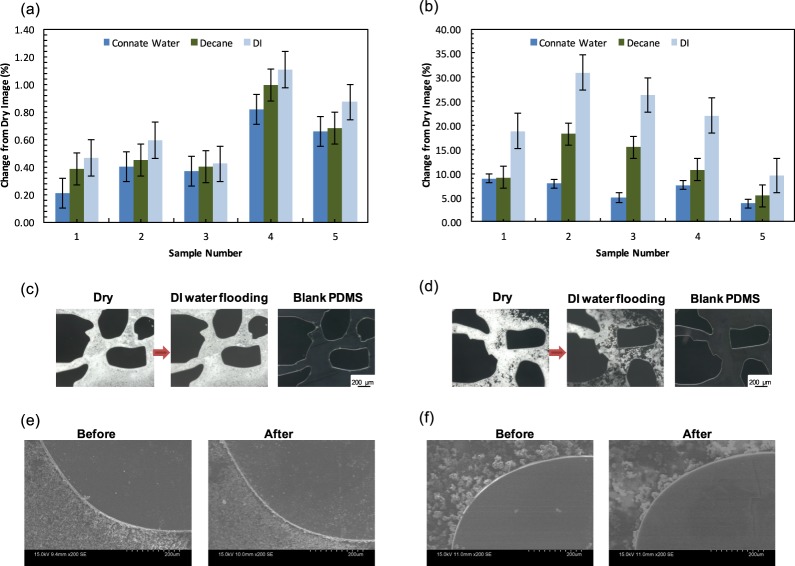
Geo-material stability measurements of (**a**) sandstone and (**b**) carbonate PDMS chips. The different injection scenarios (connate water, decane and DI) are compared to the dry image, and the percent change is shown in the column graphs. Error bars indicate the standard error of the mean. (**c**) and (**d**) show microscopic images of the highest change in both sandstone (**c**) and carbonate (**d**). The images illustrate the change from the dry image (reference) and after DI water flooding, along with a blank PDMS of the same region. As can be seen, minor change is observed in (**c**), however, the 30.9% change can be clearly visible in (**d**). Example of SEM images for dry sample (before) and after flooding for (**e**) sandstone and (**f**) carbonate.

It is essential to test the stability of the geo-material PDMS chips. In this section, we studied changes between sequential images after flooding the chip with selected fluids. Images are collected after each treatment and segmented to evaluate the amount of deposited minerals. The difference between images is reported as a percent change, as defined in the Methods section. The deposited dry chip (prior to any injection) and brine, decane, and DI water flooding scenarios in 5 different locations across 3 different chips are evaluated. Figure 10 shows the percent change between the initial condition of the geo-material PDMS chip and the subsequent flooding. From Fig. 10, the sandstone chip shows a low change across the different flooding scenarios, with the highest change being 1.1%. An average of 0.4%, 0.6% and 0.7% change between the dry image and connate, decane and DI injections, respectively. For the carbonate PDMS chips, a maximum of 30.9% change between the dry image and the subsequent flooding scenarios was measured. An average of 6.7%, 11.9% and 21.5% change between the dry image and connate, decane and DI injections where measured, respectively. In terms of particle detachment, the calcite mixture appears to detach easier than the sandstone, which is likely due to the strong siloxane bond present between the sandstone mixture and activated PDMS surfaces. The images in Fig. 10c and d provide examples of the highest change in sandstone and carbonate chips (sample 4 and 2, respectively in Fig. 10a and b) to help illustrate what 1.1% and 30.9% change looks like.

Additionally, SEM images before and after flooding are shown for each chip in Fig. 10e and f to help visualise the differences at higher resolution. As it can be depicted, the carbonate geo-material PDMS is not as stable as the sandstone. In addition, roughness measurements before (i.e. dry sample) and after the three consecutive floods was performed on 5 areas in each sample. An average change of 4.8% and 11.9% in roughness was calculated for the sandstone and carbonate geo-material chips, respectively. Overall, the microscope, SEM and roughness measurements demonstrate flooding of geo-material PDMS does show pore-level changes that result from either detachment of minerals from the PDMS surface and/or dissolution of mineral crystals. Similar mechanisms are observed in carbonate and sandstone rocks subjected to low salinity water flooding, where clay fines detach from rock surface, or dissolution of carbonate rock. Since these chips were tested using DI water (no salinity) it is likely that these mechanisms are being observed in our measurements. Both of these mechanisms are proposed oil recovery mechanisms for an enhanced oil recovery technique called low salinity flooding^68,69^. Further work is needed to relate the observed results in our geo-material PDMS to that observed in carbonate and sandstone rock under low salinity water flooding.

## Conclusions

Geo-material microfluidics approaches are essential for the visualisation and understanding of rock-fluid interactions, which are relevant to subsurface engineering applications, such as geologic CO_2_ storage, geothermal energy, enhanced oil recovery and hydrology. Thus far, we have demonstrated a simple and rapid fabrication method for a geo-material PDMS microfluidics device. We have evaluated the uniformity of the deposition using semivariograms (Fig. 3a and b), and surface roughness measurements (Fig. 3c). We then, compared our functionalised PDMS devices to their representative rock via SEM-EDS analysis (Fig. 4). From these imaging techniques, we can see that the composition and height profile in both rocks and functionalised PDMS devices are similar and that mineral deposition is nearly isotropic. The establishment of thin films with PVA treatment of the sandstone and carbonate chips (Fig. 8) have shown to be well-connected, particularly in the two PDMS chips with mineral deposition, which demonstrated that water is wetting the pore space.

In addition, we were able to capture the pore-scale flow mechanism of corner flow leading to the snap-off of the nonwetting phase in a controlled wetting environment (Fig. 8). Our fabrication method provides surface roughness and connected corners for thin-film flow. These two multiphase flow mechanisms are important for carbon capture and storage^19^ and enhanced oil recovery^5–7^ studies. Lastly, we verify the stability of our fabricated geo-material PDMS chips (Fig. 10) by measuring the similarity of chips before and after flooding with DI water and decane. When compared to the dry image of the deposited PDMS chips, the sandstone chip exhibited the highest change being 1.1%, whereas the change in the carbonate PDMS chips was 30.9%. Average roughness measurements and SEM images before and after flooding also confirm that sandstone PDMS chips are more stable than the carbonate PDMS chips. We also acknowledge that pore-level change in the attached geomaterials occur under low salinity water flooding (DI water). Future studies will include visualisation of subsurface engineering related phenomena; such as oil snap-off, enhanced oil recovery and other processes where surface chemistry is highly important, such as CO_2_ sequestration, calcite dissolution, surfactant and/or low salinity brine flooding resulting in mineral detachment and migration. Our geo-material PDMS chips can provide a useful platform to answer questions in subsurface and environmental engineering fields, in particular, when fluid-fluid and fluid-rock interactions are highly important.

## Methods

### Mineral Solution Preparation

To replicate sandstone rock, quartz and kaolinite were chosen as the dominant minerals^55^. For carbonate rock, calcium carbonate (CaCO_3_) is the major component^52^. Deionized (DI) water was used to create the following solutions: (1) 4% (w/v) Quartz (silicon dioxide, Sigma-Aldrich) and 2% (w/v) Kaolinite (Kaolin, Imerys minerals, Australia), and (2) 4% (w/v) CaCO_3_ (calcium carbonate, Normapur AR). Relatively high fractions of minerals were used to ensure a homogenous deposition on the geo-material PDMS. Solutions were mixed and sonicated (Bransonic 220) for one hour, as previously described in Song and Kovscek^47^. When mixing sandstone (Eqs 1 and 2) and calcite minerals (Eq. 3) with DI water, the following aqueous dissolutions occur:

1 $${{\rm{SiO}}}_{2}({\rm{s}})+2{{\rm{H}}}_{2}{\rm{O}}({\rm{l}})\rightleftharpoons {\rm{Si}}{({\rm{OH}})}_{4}({\rm{aq}})$$

2 $${{\rm{Al}}}_{2}{{\rm{Si}}}_{2}{{\rm{O}}}_{5}{({\rm{OH}})}_{4}({\rm{s}})+5{{\rm{H}}}_{2}{\rm{O}}({\rm{l}})\rightleftharpoons 2{\rm{Al}}{({\rm{OH}})}_{3}({\rm{aq}})+2{\rm{Si}}{({\rm{OH}})}_{4}({\rm{aq}})$$

3 $${{\rm{CaCO}}}_{3}({\rm{s}})+{{\rm{H}}}_{2}{\rm{O}}\,({\rm{l}})\rightleftharpoons {{\rm{Ca}}}^{+2}({\rm{s}})+{{\rm{HCO}}}^{-3}({\rm{aq}})+{{\rm{OH}}}^{-}({\rm{aq}})$$

### Geo-material microfluidics chip fabrication

**Figure 11:**
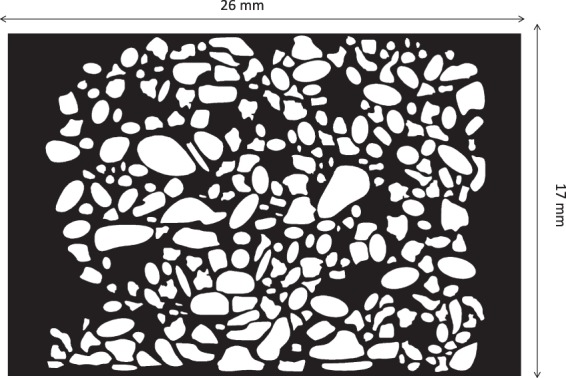
Pore network pattern of the geo-material chip. Black is the pore space and white shows the grains.

A random pore network pattern was created in AutoCAD (Version 2017, Autodesk Inc.). The morphology of the pores can be depicted in Fig. 11. The pattern was then transferred into a chrome mask for silicon mould microfabrication using standard lithography techniques, as previously described^70,71^. The silicon mould was etched using deep reactive ion etching (DRIE) to define the channel features on the wafer to achieve an etch depth of 100 µm. The process of creating the silicon mould was outsourced to the South Australian node of the Australian National Fabrication Facility. This mould was then silanized, coated with de-gassed PDMS (Sylgard 184, Dow Corning Corporation) at ambient conditions and then cured for 4 hours at 65 °C in-house using a well-known process termed “soft lithography”^72^. The resulting PDMS slice containing the porous media was then peeled off the silicon mould and treated with oxygen plasma (Harrick Plasma Cleaner, NY, USA) for 4 minutes and 15 seconds, and 1 ml of the desired mineral solution was immediately placed on top of the PDMS slice. The PDMS slice was then air-dried and cleaned with ethanol and inspected using a stereo microscope (PSB X2-4, Saxon) to ensure no particles are present in the top of PDMS pillars. Lastly, a blank PDMS cover was bonded to the PDMS slice containing the porous medium with the deposited mineral via a second plasma treatment. The reason why the PDMS cover is left blank because the silanol groups on the PDMS are required for permanent bonding between the cover and the pillars of the PDMS with minerals. Examining the PDMS prior to the second plasma treatment was crucial for strong bonding of the porous media containing the minerals and the PDMS cover. If some particles were present on the top surface of the PDMS pillars, this would result in weak bonding. Figure 2 summarises the fabrication workflow. The total time needed to fabricate the geo-material PDMS microfluidic chip was 4.5 hours.

### Characterisation of the Geo-material Microfluidic system

Semivariograms are used to study spatial arrangement of minerals after deposition. This is done by evaluating the variations in the pixel intensities of the grey-level values across stereo microscope images fabricated chips^73,74^. The semi-variance $\gamma (h)$ is described as

4 $$\gamma (h)=\frac{1}{2N(h)}\,\sum _{i=1}^{N(h)}{[Z({x}_{i})-Z({x}_{i}+h)]}^{2}$$

where *h* is the lag distance (or pixel distance), $Z({x}_{i})\,and\,Z({x}_{i}+h)$ are the pixel intensity values in the image, *Z* at a separation of *h* and $N(h)$ is the number of pixel pairs at a lag distance, *h*. The anisotropic nature of an image can be captured by constructing semivariograms in different directions^75^. The semi-variance for different lag distance in both horizontal (0°) and vertical (90°) directions can be calculated. If the semi-variances in these directions are similar, the image is isotropic. To test the isotropic distribution of minerals deposited on the PDMS chips, we conducted semivariogram analysis on three carbonate and three sandstone mineral deposited PDMS chips. Before analysis, images representing the deposited minerals were isolated from the PDMS grains while maintaining their spatial locations to ensure that only the semivariance of deposited minerals are evaluated rather than the PDMS porous pattern. Then, the original 8-bit image was re-quantised to a 6-bit image using uniform quantisation^76^. Lastly, each of these images were cropped to a size of 1600 × 1600 to ensure that the number of pixel pairs for varying lag distances remained the same in both horizontal (0°) and vertical (90°) directions.

Surface roughness of coated microfluidics chips and their respective rock slices were measured using a 3D laser microscope (VK-X200, Keyence), as previously described in Gerami, *et al*.^51^. Rock slices were 30 mm in diameter and 3 mm height thin sections, which were cut using a saw and were put in a vacuum oven at 65 °C to dry for 24 hours. It is noteworthy that the rocks were unpolished. The microscope has a resolution of 0.5 nm and 1 nm in the z direction and X-Y plane, respectively. The magnification of the microscope was set at 400× for the measurements. The measured roughness of the geo-material microfluidics devices was compared against slices of their respective rock types. Roughness is measured based on the arithmetic mean roughness, which indicates the average of the absolute value along the reference length^77^. Eq. 5 refers to the average elevation (*Z*_*c*_), which is the average value of elevation of each curve element (*Z*_*ti*_) on the reference length. Eq. 6 states the average elevation along the X-Y plane, with *Z*_*t(xi,yj)*_ as the elevation in the X and Y direction, respectively.

5 $${Z}_{c}=\,\frac{1}{m}\sum _{i=1}^{m}{Z}_{ti}$$

6 $${Z}_{c}=\,\frac{1}{mn}\sum _{i=1}^{m}\sum _{j=1}^{n}{Z}_{t(xi,yj)}$$

where *m* and *n* indicate the total number of curved elements in the X and Y directions, respectively.

Lastly, the rock slices and PDMS chips were coated with ~20 nm carbon and imaged by scanning electron microscopy (SEM, Hitachi, S-3400N) coupled with energy dispersive X-ray spectroscopy (EDS detector, Bruker X-Flash 5010) analysis at 15 kV. SEM-EDS analysis was done to identify the minerals and elements present on the coated PDMS chip compared to their respective rock types.

### Geo-material PDMS Chips Wettability Alterations

To replicate sandstone and carbonate rock properties for the geo-material microfluidic PDMS chip, the wettability conditions must be controlled. PDMS is naturally oil-wet, and with oxygen plasma exposure it becomes water-wet for a maximum of 2 hours^59,61^. Trantidou, *et al*.^61^ developed a method to modify the wettability of the surface of PDMS to water-wet with the addition of polyvinyl alcohol (PVA) immediately after oxygen plasma. Water-wet sandstone, carbonate and blank PDMS chips were treated with oxygen plasma and put in conformal contact to allow bonding between the sandstone and carbonate chips with the blank PDMS cover. These chips were immediately injected with PVA to fill the entire microfluidic chip via a plastic syringe and left for 10 minutes at room temperature. The PVA was then removed with injecting air in the microfluidic chips and heated to 110 C° for 15 minutes. Details on this method is described in Trantidou, *et al*.^61^. After PVA treatment, the chips were left for 3 days to eliminate the effect of the oxygen plasma treatment. The chips were then injected with brine of 10% NaCl and 2% (w/v) Green food colour (AmeriColor, Placentia, CA, USA) for multiple pore volumes, followed by decane (Sigma Aldrich, USA) injection to achieve 80–90% oil saturation and left 20 minutes to equilibrate. Images of the sandstone, carbonate and blank PDMS chips were taken after three days with and without PVA treatment using a stereo zoom microscope (Zeiss, Axio Zoom V16) to characterise surface wettability with and without PVA treatment. From the collected microscopic images, the PDMS chips were characterised as either water-wet or oil-wet depending on the pre-dominant fluid (oil or water) surrounding the grains of the PDMS chips.

### Contact Angle Measurements

Contact angle measurements were taken on blank PDMS chips and the different geo-material PDMS chips along with their respective rock types with and without PVA treatment. This is done to assess the wettability of the fabricated chip in comparison to their equivalent rock types. The contact angles are for air/DI-water/rock or PDMS chips. A droplet of deionized water was placed on the surface of a sample and contact angle was measured from the collected image using the contact angle plugin for ImageJ^78^. To validate contact angles for the sandstone and carbonate geo-material chips (with and without PVA), the Cassie’s equation^63,64^ was used and effective contact angles were measured as

7 $$\cos \,{\theta }_{eff}={f}_{Gm}\,\cos \,{\theta }_{Gm}+{f}_{PDMS}\,\cos \,{\theta }_{PDMS}$$

where ${\theta }_{eff}\,$is the effective contact angle, ${f}_{Gm}\,$is the fraction of the geomaterial (minerals) on the PDMS surface, ${\theta }_{Gm}$ contact angle of the geo-material, ${f}_{PDMS}$ fraction of PDMS and ${\theta }_{PDMS}$ is the PDMS contact angle. Fractions of both PDMS and minerals (${f}_{PDMS}\,$and ${f}_{Gm}$, respectively) were measured based on segmented images of the same PDMS coated mineral surfaces that were used previously for experimental contact angle measurements.

### Flow Visualisation

To reproduce fluid distributions within a natural reservoir, the chips were first injected with brine of 10% NaCl (w/v) and 2% green food colour (w/v) (AmeriColor, Placentia, CA, USA), and then decane (Sigma Aldrich, USA) was injected to achieve a 80–90% oil saturation and left 20 minutes to equilibrate. This step establishes connate water saturation within the chip. Then, DI water with 2% red food colour (w/v) (AmeriColor, Placentia, CA, USA) was injected at a capillary number (*Ca*) of 10^−5^ to replicate water flooding conditions. A high-precision infusion syringe pump (Fusion 100 Infusion, Chemyx, Australia) is used to monitor and control the flow rate (4 µm/min). Optical microscope images were collected using a CCD digital camera (Zeiss, Axiocam 506 colour) coupled with a stereo zoom microscope (Zeiss, Axio Zoom V16) with a size of 2752 × 2208 pixels. All the experiments were conducted at ambient conditions. The Young-Laplace equation, which relates the mean curvature of the fluid-fluid interface to capillary pressure (*P*_*c*_) was used to study the snap-off event. Differences in local capillary pressure are known to result in snap-off under specific criteria^67,79,80^. Capillary pressure (*P*_*c*_) is calculated as

8 $${P}_{c}=\sigma (\frac{1}{{R}_{1}}+\frac{1}{{R}_{2}})$$

where *σ* is interfacial tension (N/m) and *R*_1_ and *R*_2_ are the principal radii of curvatures for the oil/water interface (m). The principal radii of curvature (R_1_) of the fluid/fluid interface that is visualised in the focal plan can be directly measured from the image^67^. The second principal radii (R_2_) of curvature is estimated based on the form of the interface. If the interface is hyperboloid, then R_2_ can be directly measured from the image as half the width of the hyperboloid at its narrowest constriction. For the case of the interfaces at the front and end menisci of an oil cluster, R_2_ cannot be directly measured from the collected images and it is assumed to be half of the microfluidics device depth. A similar assumption is made in previous publications^80–82^. Due to the complexity in measuring contact angle for the functionalised surface, there is no direct way to actually measure the curvature in the plane orthogonal to the focal plane, and thus, we assume complete wetting conditions. This assumption is only an order of magnitude estimate that allows for evaluation of capillary pressure difference over the nonwetting phase.

### Minerals Attachment Stability of the Geo-material PDMS chips

**Figure 12:**
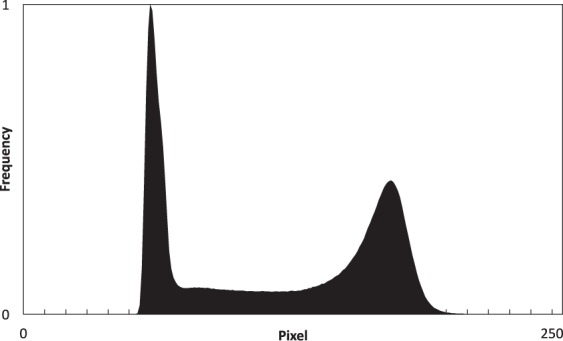
An example histogram of an 8-bit greyscale image used in the stability studies.

To test the stability of the mineral deposition, we measured the degree of mineral detachment from the geo-material chip after sequential flow experiments. Five locations were chosen from three samples of sandstone and carbonate PDMS chips, and imaged using a stereo zoom microscope (Zeiss, Axio Zoom V16) after the following conditions: (1) dry (no prior to injection), which is considered as the reference sample, (2) connate water (10% NaCl (w/v)) injection (3) decane injection, and (4) DI water injection. All the images in the different injection conditions were obtained after 4–5 PV of injected fluid at a rate of 5 μl/min. The samples were dried in a vacuum oven at 65 °C for 25 minutes after each injection to obtain a clear image, free of liquid. Images were converted to 8-bit greyscale, which gave a bi-modal histogram of the intensity values that ranged from 0 to 256 An Example histogram is shown in Fig. 12, which displays a clear separation of intensity values between deposited minerals and PDMS.

The images were then segmented at a unified pixel value of 115 to identify the surface minerals. This value was defined based on the Auto Threshold function in ImageJ, which uses the IsoData method for thresholding^83^. The sum of pixels was estimated for the entire thresholded image after each flooding using ImageJ. The net percentage change between each flooding scenario and dry image was measured as

9 $$ \% \,{\rm{Change}}=\frac{{P}_{dry}-{P}_{Inj}}{{P}_{dry}}\times 100$$

where *P*_*dry*_ is the sum of pixel values of the dry geo-material PDMS and *P*_*inj*_ is the sum of pixel values in the geo-material PDMS after the different injection scenario. We also provide grey-scale images before and after treatment to illustrate the sensitivity of the calculation presented in Eq. 9. To allow for better quantification and visualisation of the changes occurring from the dry geo-material PDMS and the above-mentioned floods (connate water, decane and DI water), surface roughness was measured (as described previously) and SEM images (Hitachi, S-3400N) were taken for the dry chip and after drying the sample from the final flooding scenario (DI water).
